# Field validity and spatial accuracy of Food Standards Agency Food Hygiene Rating scheme data for England

**DOI:** 10.1093/pubmed/fdaa172

**Published:** 2020-09-24

**Authors:** Scott Kirkman, Bruce Hollingsworth, Amelia Lake, Stephanie Hinke, Stewart Sorrell, Thomas Burgoine, Heather Brown

**Affiliations:** Population Health Sciences Institute, Newcastle University, and Fuse -Centre for Translational Research in Public Health, UK; Lancaster University, UK; Teesside University, and Fuse -Centre for Translational Research in Public Health, UK; University of Bristol, Erasmus University Rotterdam, Institute for Fiscal Studies, UK; (Health and Safety) Communities and Environment, Gateshead Council, UK; UKCRC Centre for Diet and Activity Research (CEDAR) and MRC Epidemiology Unit, University of Cambridge, UK; Population Health Sciences Institute, Newcastle University, and Fuse -Centre for Translational Research in Public Health, UK

**Keywords:** food environment, foodscape, field validity, spatial accuracy

## Abstract

**Background:**

The study aimed to evaluate the validity and spatial accuracy of the Food Standards Agency Food Hygiene Rating online data through a field audit.

**Methods:**

A field audit was conducted in five Lower Layer Super Output Areas (LSOAs) in the North East of England. LSOAs were purposively selected from the top and bottom quintiles of the Index of Multiple Deprivation and from urban and rural areas. The FHRS data validity against the field data was measured as Positive Predictive Values (PPV) and sensitivity. Spatial accuracy was evaluated via mean difference in straight line distances between the FHRS coordinates and the field coordinates.

**Results:**

In all, 182 premises were present in the field, of which 162 were in the FHRS data giving a sensitivity of 89%. Eight outlets recorded in the FHRS data were absent in the field, giving a PPV of 95%.The mean difference in the geographical coordinates of the field audit compared to the FHRS was 110 m, and <100 m for 77% of outlets.

**Conclusions:**

After an evaluation of the validity and spatial accuracy of the FHRS data, the results suggest that it is a useful dataset for surveillance of the food environment and for intervention evaluation.

## Introduction

The food environment is thought to play a role in obesity rates and other chronic health conditions.^1^^,^^2^ Proximity to fast food outlets is associated with increased obesity, particularly for children.^3^ Lifestyle and diet contribute to observed socioeconomic differences in weight and obesity.^4^^,^^5^ There are also important health inequalities around access to healthier food.^6^^,^^7^ Unhealthy food outlets tend to cluster in more socially deprived areas and those from lower socioeconomic status groups are more vulnerable to the resulting detrimental effects on diet and health.^8^^,^^9^ Given the associations between the food environment and health, researchers have expressed a need for longitudinal data to assess the impact of changes to the food environment.^10^

In England since 2013, local authorities (LAs) have responsibility for public health including oversight of obesity, community nutrition and promoting physical activity.^11^^,^^12^ An umbrella review by Public Health England assessed the importance of the built and natural environment on health.^13^ The review identified key areas that can be targeted by LAs through policy, two of which were access to healthy food and neighbourhood design. Given their public health obligations, many LAs have taken an active role in managing the food environment, often through the use of planning policy to limit the number of new takeaway food outlets.^8^^,^^11^

LAs in England can influence the proliferation of food establishments with planning policies described in local plans and planning guidance.^8^^,^^11^ Around 50% of LAs have a planning policy to control numbers of takeaway and fast food outlets. Much of the planning guidance employed by LAs restricts the number of new outlets within a geographical area such as within 400 m of a school. Thus, it is important to understand how these geographical restrictions shape the food environment. ^6^^,^^9^ Despite their widespread uptake, there is currently no evidence regarding the effectiveness of these LA restrictions on improving health or reducing health inequalities.

The lack of evidence on the effectiveness of these planning policies may result from insufficient or inadequate data pertaining to the neighbourhood food environment and locations of food outlets. Examples of data sources used in food environments research to date include commercially available business data such as the Yellow Pages, data from companies such as Ordnance Survey, company websites or Google Maps and data obtained from local government under Freedom of Information requests.^1^^,^^4^ Limitations of these data sources include lack of national coverage, resource intensive data collation, infrequent updates, restrictive terms and conditions of use and financial cost.^14^ While there are many field validation studies of commercially available business data,^11^ few studies consider the spatial accuracy of data on the food environment. Analysis of commercial data in Canada found 75% of food outlets in the field were within 50 m of their recorded location in the data.^15^ Similar methods have also been used to assess the accuracy of physical activity facilities data in the USA, motivated by the association between access to play parks and health.^16^

This study aims to assess the validity and spatial accuracy of the Food Standards Agency (FSA) Food Hygiene Rating Scheme (FHRS) data using field validation methodology in the North East of England. Field validation involved a researcher recording the business name and geographical coordinates of all business premises selling food in the selected field work locations. These field data are then treated as a gold standard for comparison against the FHRS data. An understanding of the validity and spatial accuracy of the FHRS data will help determine whether the dataset can be used for evaluation and surveillance of the food environment by public health teams and researchers.

## Methods

### The data

Since 2012, data collected by environmental health officers for the FSA FHRS including the geographical coordinates of all businesses/premises where food is consumed, sold or provided for all LAs in England, Scotland, Wales and Northern Ireland have been available online. This includes restaurants, pubs, cafes, takeaways, food vans/stalls, schools, canteens, hotels, supermarkets and other shops (e.g. garage forecourt shops, convenience stores), care homes, community centres, cafes within retail shops, nurseries and hospitals. All new businesses need to be registered with the LA at least 28 days before opening. All LAs need to upload data of recently inspected premises at least every 28 days. Frequency of inspection depends upon the hygiene rating received by the business/premises.

These data are national in coverage, available for public download, updated regularly, unrestricted in terms of use and free.

Sample data were extracted from the FSA website on 4 October 2019.

As this study used publicly available data, which are not covered under GDPR legislation, ethical approval was not required.

### Study areas

Study areas were selected purposively as in Lake *et al.*^4^ across two LAs to include areas of high and low deprivation as defined by the Index of Multiple Deprivation and classified as either urban and rural using the 2011 Rural–Urban Classification of LA districts.^17^^,^^18^ The LAs of Gateshead and Northumberland were chosen in the North East of England for the convenience of access for the researcher. Gateshead LA has actively attempted to manipulate the food environment through using planning guidance to restrict the conversion of any existing premises to planning use Class A5—hot food and takeaway.^19^ Northumberland implemented planning guidance in March 2020 (after the field audit was conducted), to limit the conversion of premises to takeaways in high-density areas or where there is high obesity prevalence in the population.^20^

Within each LA four study areas were selected based on Lower Layer Super Output Area (LSOA) boundaries. LSOAs are geographical areas which contain on average 1500 people, but vary in size.^21^ One urban and one rural LSOA from the top and the bottom index of multiple deprivation quintiles for each LA were selected for a field audit.^17^^,^^18^ Of the 128 eligible LSOAs, we chose to audit the LSOA with highest recorded number of outlets in the FHRS data to improve the statistical precision of our findings’.^4^ The eight selected study area LSOAs are shown in Table 1. Unfortunately, the Covid-19 Coronavirus pandemic occurred during the fieldwork period and it was not possible to conduct fieldwork in the final three study areas denoted in grey. We were unable to assess validity in three LSOAs in Northumberland two urban (least and most deprived) and one rural (least deprived).

**Table 1:** Target fieldwork LSOAs, characteristics and number of recorded outlets

Local authority	Rural/Urban ^a^	Deprivation ^b^	Outlets	People per sq km^c^
Gateshead	Rural	Most	14	1424
Gateshead	Urban	Most	82	1523
Gateshead	Rural	Least	12	1074
Gateshead	Urban	Least	19	5232
Northumberland	Rural	Most	43	1267
Northumberland	Urban	Most	76	3325
Northumberland	Rural	Least	45	82
Northumberland	Urban	Least	65	503

a 2011 Rural–Urban Classification of Local Authority Districts in England

b The English Indices of Deprivation 2019

c Based on 2018 population estimates

### Field audit

A field audit was conducted to ‘ground-truth’ targeted geographic areas, recording all potential premises selling food in the chosen LSOAs. For the field audit, a field work form was developed before undertaking the audit using the FHRS data and contained information on all premises in the LSOA including address and geographical coordinates. This form was created to avoid less visible premises (such as food businesses run from household kitchens) from being missed in the field.

In each area a systematic field audit was conducted on foot, using a mobile GPS app–Locus Map, to record the details and coordinates of the outlets.^22^ One researcher (SK) recorded all possible food outlets that were present in the field directly in the app and on paper fieldwork forms (example available in the appendix) so that while conducting the audit the researcher had knowledge of which premises should be present. The latitude and longitude of the researcher position was recorded automatically by the app. Outlets present in the field and in the FHRS data were recorded alongside their FHRS ID, and outlets present only in the field were allocated a new ID. The app allowed for all of the data to be recorded digitally and later exported as a CSV file, while paper fieldwork forms were also completed in case of data loss.

### Validation and spatial accuracy

The data collected during the field audit was compared with data in the FHRS dataset using a field validation method which has been used previously in other field validation studies.^1^^,^^4^ This was to assess any discrepancies between the field audit and FHRS data. The validity outcome measures were Predicted Positive Value (PPV) and sensitivity. They were calculated as below:

(1) \begin{equation*} \mathrm{PPV}=\mathrm{True}\ \mathrm{Positive}/\left(\mathrm{True}\ \mathrm{Positive}+\mathrm{False}\ \mathrm{Positive}\right) \end{equation*}

(2) \begin{equation*} \mathrm{Sensitivity}=\mathrm{True}\ \mathrm{Positive}/\left(\mathrm{True}\ \mathrm{Positive}+\mathrm{False}\ \mathrm{Negative}\right) \end{equation*}

Where True Positive is the number of outlets present in both the secondary data and the field data, False Positive is the number of outlets present in the secondary data but absent in the field, and False Negative is the number of outlets present in the field but absent in the secondary data.

To assess the spatial accuracy of the geographical coordinates in the FHRS data, straight-line distances were calculated between the FHRS coordinates and the field location of each outlet in Stata v.16 using the geodist package.^15^^,^^16^^,^^23^ Given the skewed nature of the distances and limited sample size, non-parametric testing in the form of Wilcoxon Rank-Sum tests were employed to formally assess differences in the distributions across rural/urban and deprivation at a 95% significance level between the field audit and FHRS data.

## Results

### P‌PV and sensitivity

The comparison of the field and FHRS data is shown in Table 2. In all, 182 premises were present in the field, of which 162 were in the FHRS data and 20 absent, giving a sensitivity of 89%. Eight outlets recorded in the FHRS data were absent in the field, giving a PPV of 95%.

**Table 2:** FHRS data and field data comparison

	Field		
FHRS	Present	Absent	
Present	162	8	PPV = 95%
Absent	20		
	Sensitivity = 89%		

### Spatial accuracy

**Fig. 1:**
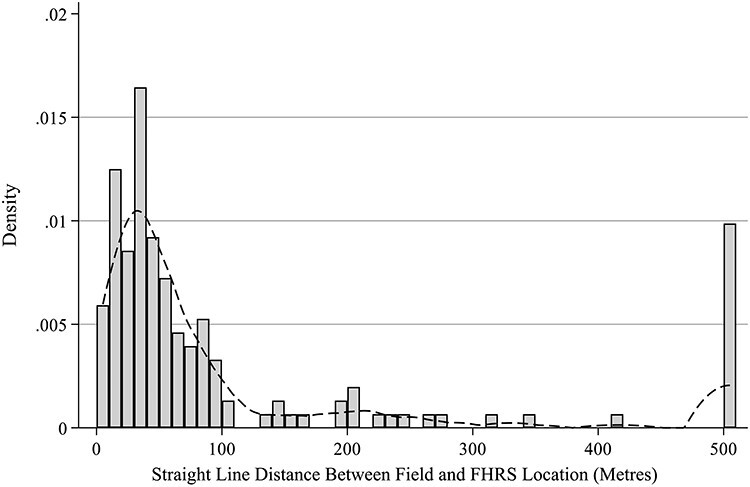
Straight-line distances between field and FHRS coordinates.

Of the 162 premises present in both the field and the FHRS data, nine had no recorded coordinates in the FHRS data. Distances between the field coordinate and FHRS coordinates were calculated for the remaining 153 premises. There was one outlier, with a distance of 37 870 m, which was excluded from the analysis. The spatial accuracy for the 152 premises is shown in Fig. 1 and the distances ranged from 5 to 505 m. The distribution had a long right tail with a median of 48 m and mean 110 m. The distance was <100 m for 117 (77%) of outlets. There was a cluster at 505 m, as 15 outlets (predominantly mobile caterers offering food to takeaway) shared the same physical site and FHRS coordinates, and therefore the distance between the FHRS coordinates and field work coordinates was the same for these 15 premises.

**Fig. 2:**
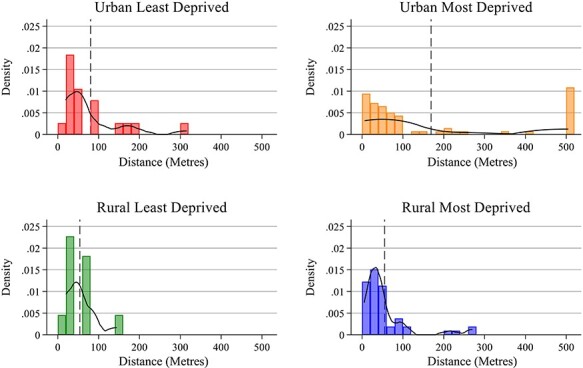
Straight-line distances between field and FHRS coordinates by urban/rural area level deprivation.

Figure 2 shows the distances split by rurality and deprivation. Rank-sum tests found no difference in the distribution of distances between most and least deprived LSOAs (*P* = 0.944). The distances were significantly smaller for outlets in rural LSOAs (*P* = 0.002). However, if the 15 premises which share the same location are treated as one premises, the difference in distance between the FHRS data and field work data is no longer significant at a 95% level (*P* = 0.078).

## Discussion

### Main finding of this study

This study assessed the validity and spatial accuracy of the FSA FHRS data, which are publicly available, national in coverage and updated regularly. A field audit of a purposive sample was conducted in the North East of England. The field audit identified 182 premises in five LSOAS. In all, 162 of these premises were present in the FHRS data. When comparing the field audit data to the FHRS data, the PPV was 95% and sensitivity was 89%.

The 20 outlets that were not identified in the data source were churches, community centres and a caravan park that was not clear if it was operational. It is possible that these establishments do not regularly serve food and would be excluded from the data. However, to be on the cautious side, we included these premises in the field work. Thus, our estimates would be a lower bound of the accuracy of the data.

The mean spatial inaccuracy of the FHRS data compared with the field audit was 110 m, 77% of outlets were within 100 m of their recorded location and all were within ~500 m—with the exception of one outlier. Spatial accuracy was greater in rural LSOAs, however this finding is sensitive to the inclusion of one urban site which is shared by 15 premises with the same FHRS coordinates. If this location with the 15 outlets was removed from the data, there was no significant difference between urban and rural spatial accuracy.

### What is already known on this topic

While LAs are active in their attempts to manage the food environment,^9^ researchers have largely been using either high cost field audits or data from commercial directories to study the food environment.^1^ These data sources are problematic due to a lack of national coverage, resource intensive data collation, infrequent updates, restrictive terms and conditions of use and financial cost.^14^ A systematic review of 20 validity studies of commercially available business data on the foodscape reported average median PPV scores of 77% and sensitivity at 60% (for the four UK studies median PPV = 81% and Sensitivity = 61%).^1^ The highest reported median PPV in the UK studies reviewed was 87% and the highest median sensitivity 87%, however, these relate to separate data sources. The highest PPV score of 87% also does not strictly pertain to commercially available business data but instead to LA data, akin to the FHRS data, obtained via a Freedom of Information request.^4^

In the USA, the accuracy of a commercial dataset of community-level physical activity facilities has previously been conducted, calculating distances from field locations and their recorded position in the commercial data.^16^ They found ~71% of facilities to be on the same street. In Canada, a study assessed the spatial accuracy of two commercially available datasets of food outlets, and found that 75% were within 50 m of their recorded positions.^15^ When comparing our results to those from other field validation studies, this would suggest that at least for the North East of England, the FHRS should be a valuable tool for evaluation and surveillance of the food environment by public health practitioners and researchers.

### What this study adds

This study is the first field validation of the FHRS data which have been available digitally since 2012. In terms of validity, the results indicate that FHRS data are likely to be superior to commercially available business data or LA data previously assessed in the UK—PPV 95% versus 87%, and Sensitivity 89% versus 87%.^4^ We find a high validity of the FHRS data, most likely because it is a statutory requirement for LAs to collect these data, suggesting it should accurately reflect the current food environment.

This study is also the first to assess spatial accuracy of food environment data in the UK, using methods from studies in the USA and Canada.^15^^,^^16^ Around 75% of outlets in the FHRS data were within 100 m, however, there were also outlets with missing coordinates in the data and one clear outlier. This suggests that the FHRS data may be slightly less spatially accurate than commercially available data in Canada, where 75% of outlets were within 50 m.

Given the high time cost to ground-truth even small geographical areas, the FHRS data will be a valuable resource for spatial analysis with caveats around exact accuracy depending upon the research question. Researchers should be aware of occasional missing coordinate data, and the potential for error in the coordinate data. As ~50% of LAs in England use planning guidance to promote a healthy food environment and much of this guidance limits new takeaway outlets within a pre-defined geographical area, this shows that there will be an interest by public health practitioners for a cost-effective means to evaluate the effectiveness of these interventions to promote a healthier environment.

There is also archived historic FHRS data, which enable researchers to build longitudinal panels to study the food environment over time—starting in 2012. With this historic data, researchers can create datasets that allow for causal inference by exploiting natural experiments, such as changes in planning policy. This dataset may be valuable for post Covid-19 research to understand how the food environment has changed as a result of the pandemic and how that may impact on health outcomes and inequalities.

### Limitations

Ideally, fieldwork by more than one researcher would have been conducted over a larger area allowing for PPV and sensitivity to be evaluated across a larger geographical area. Unfortunately, the Covid-19 pandemic resulted in the cancellation of some intended field audits. Thus, we were not able to assess and compare accuracy between urban areas in the two LAs of Gateshead and Northumberland as the data were limited to urban Gateshead only.

The study sample was also limited to the North East of England, which can be seen as a limitation. However, due to the statutory nature and requirements for data collection which will mean that data will be presented and collected similarly nationally. Even though the UK is heterogenous in terms of urbanity and density of outlets, validity and spatial accuracy should be similar to what we found in the North East of England. To test this assumption, it may be worthwhile to do further validation checks of the data in other parts of the UK. However, for a purposive sample from the North East, the selection of LSOAs with the highest density of outlets maximized sample size for a fixed number of study areas.

Finally, it is possible that non-public facing outlets, which were not in the FHRS data, may have been missed in the field. This would inflate the sensitivity estimates.

However, it is a legal requirement for outlets to register with the FSA and therefore this should not be a substantial issue. Conversely, the sensitivity estimate may also be an underestimation as we mentioned above—the fieldwork researcher recorded all possible premises which may serve food, including churches and community centres, some of which may not serve food on a regular basis and therefore would not be inspected and present in the data.

## Conclusion

This study demonstrates that the FHRS has high validity and spatial accuracy in the North East of England. This suggests that the FHRS data may be a valuable resource for evaluation of public health interventions to promote a healthy food environment and for surveillance of the food environment.

## Funding

This article presents independent research funded by the NIHR School for Public Health Research (SPHR). The views expressed are those of the author(s) and not necessarily those of the NHS, the NIHR or the Department of Health and Social Care.

T.B. is funded by the Centre for Diet and Activity Research (CEDAR), a UK Clinical Research Collaboration (UKCRC) Public Health Research Centre of Excellence. Funding from the British Heart Foundation, Cancer Research UK, Economic and Social Research Council, Medical Research Council, the National Institute for Health Research [grant number ES/G007462/1], and the Wellcome Trust [grant number 087636/Z/08/Z], under the auspices of the UK Clinical Research Collaboration, is gratefully acknowledged.

## Notes

Scott Kirkman, Research Assistant

Bruce Hollingsworth, Professor of Health Economics

Amelia Lake, Professor of Public Health Nutrition

Stephanie von Hinke, Professor of Economics

Stewart Sorrell, Team Leader

Thomas Burgoine, Senior Research Associate

Heather Brown, Senior Lecturer

## Appendix A1

### Example fieldwork form

**Table:**

**FHRS ID**	**Business name**	**Business type**	**Address line 1**	**Outlet type**	**Field latitude**	**Field longitude**
812074	Newbiggin Convenience Store	Retailers—other	104 Front Street			
288723	Glentons Bakery	Retailers—other	108–110 Front Street			
…	…	…	…			

An example of the tables used on the paper fieldwork forms to record outlets and their locations. The table contained all of the FHRS outlets for the given LSOA as well as additional rows for outlets present in the field but not in the data.
